# SRAS‐net: Low‐resolution chromosome image classification based on deep learning

**DOI:** 10.1049/syb2.12042

**Published:** 2022-04-04

**Authors:** Xiangbin Liu, Lijun Fu, Jerry Chun‐Wei Lin, Shuai Liu

**Affiliations:** ^1^ Hunan Provincial Key Laboratory of Intelligent Computing and Language Information Processing Hunan Normal University Changsha China; ^2^ College of Information Science and Engineering Hunan Normal University Changsha China; ^3^ Hunan Xiangjiang Artificial Intelligence Academy Changsha China; ^4^ Department of Computer Science, Electrical Engineering and Mathematical Sciences Western Norway University of Applied Sciences Bergen Norway

**Keywords:** chromosome classification, low‐resolution chromosome, self‐attention negative feedback network, SMOTE

## Abstract

Prenatal karyotype diagnosis is important to determine if the foetus has genetic diseases and some congenital diseases. Chromosome classification is an important part of karyotype analysis, and the task is tedious and lengthy. Chromosome classification methods based on deep learning have achieved good results, but if the quality of the chromosome image is not high, these methods cannot learn image features well, resulting in unsatisfactory classification results. Moreover, the existing methods generally have a poor effect on sex chromosome classification. Therefore, in this work, the authors propose to use a super‐resolution network, Self‐Attention Negative Feedback Network, and combine it with traditional neural networks to obtain an efficient chromosome classification method called SRAS‐net. The method first inputs the low‐resolution chromosome images into the super‐resolution network to generate high‐resolution chromosome images and then uses the traditional deep learning model to classify the chromosomes. To solve the problem of inaccurate sex chromosome classification, the authors also propose to use the SMOTE algorithm to generate a small number of sex chromosome samples to ensure a balanced number of samples while allowing the model to learn more sex chromosome features. Experimental results show that our method achieves 97.55% accuracy and is better than state‐of‐the‐art methods.

## 1 INTRODUCTION

Chromosomes are important carriers for the transmission of genetic information. Healthy humans have 22 pairs of autosomes and one pair of sex chromosomes (male XY and female XX). The different chromosomes differ in length, centromere position and band type [1]. In medicine, karyotype analysis is commonly used to diagnose whether the foetus has congenital diseases, such as trisomy 21. In clinical practice, doctors usually collect the cells in the amniotic fluid of pregnant women, capture the chromosomes in the metaphase of cell division by cell culture, and then use quinacrine dihydrochloride, Giemsa, or other staining techniques to assign light and dark bands to the chromosomes [2]. Q‐banded chromosomes are the chromosomes stained with quinacrine dihydrochloride. After that, the physician segments each chromatid from the captured cell image. Based on the characteristics of each chromosome, the physician sorts the chromosomes according to the international standard format and creates a karyotype analysis chart [3]. From the karyotype analysis chart, doctors can obtain diagnostic information such as physical defects and genetic diseases of the foetus. These diseases can be assessed based on the number of each chromosome type and the deletion, duplication, inversion or ectopy in the structure [4]. In traditional karyotype analysis, doctors need to manually segment and classify chromosomes by careful observation with the naked eye to create the karyotype analysis maps. This takes at least a week and depends greatly on the doctor's professional knowledge.

In order to reduce the burden on doctors and save the consumption of resources such as manpower and materials, some experts and scientists have proposed an automatic or semi‐automatic procedure for karyotype analysis. In the early years, machine learning methods were used to extract the feature vectors of different chromosome types and then calculate the distance between the unknown chromosome and each chromosome type to determine which chromosome type the chromosome belongs to [5, 6, 7, 8]. With the rapid development of deep learning in recent years, the use of deep learning for chromosome classification has achieved good results, but there are still some challenges.

**FIGURE 1:**
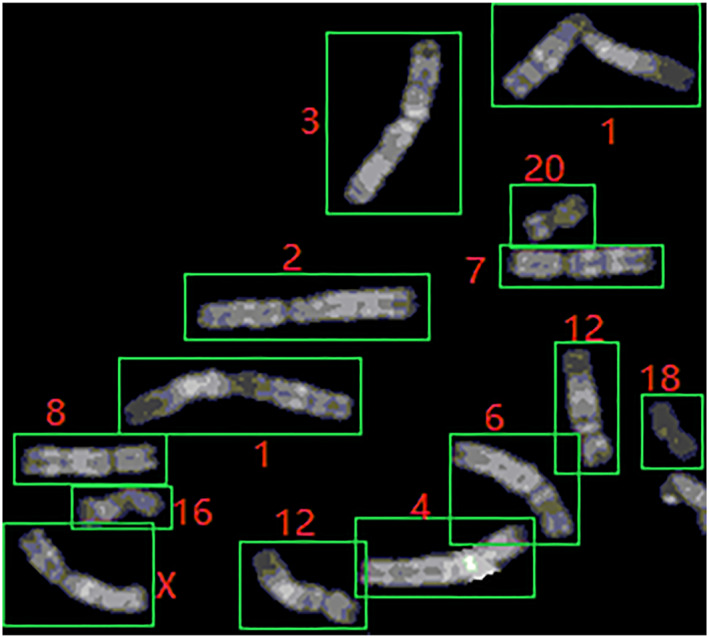
Chromosomes 1, 2, and 3 and chromosomes X, 6, 7, and 8 have a high degree of similarity in terms of length and banding distribution

According to previous studies [9, 10, 11, 12], the challenges of using deep learning for chromosome classification mainly arise from two aspects: data similarity and imbalance. First, in the metaphase chromosome image, there are some similarities in the length and banding of some chromosomes. In medicine, chromosomes can be divided into seven categories according to their length: A, B, C, D, E, F, and G [13]. Thus, there are different types of chromosomes that are similar in length. Moreover, the resolution of the photographed chromosomes is low due to hardware equipment, which causes the striped features of q‐band chromosomes of different classes to be similar. This can be observed in Figure 1. Since these two features are important parameters in chromosome classification, the occurrence of the above two problems makes chromosome classification difficult. Second, in chromosome diagnosis, physicians often extract chromosomes from the whole cell image. In a cell, there are 22 pairs of autosomes and one pair of sex chromosomes (male XY and female XX), which inevitably results in the X and Y chromosomes in a chromosome library being much fewer than other autosomes. Since the satellite features of the Y chromosome are obvious, it is less affected by this problem. However, the X chromosome is very similar to No. 6–12 autosomes. In this case, the above problems directly cause the model to be not able to learn the features of X chromosome well, so the recognition effect of X chromosome is not good.

In response to the above problems, in this paper, we propose an SRAS‐net method for low‐resolution chromosome image classification. First, we propose to use Self‐Attention Negative Feedback Network (SRAFBN) [14] to convert low‐resolution (LR) chromosome images into high‐resolution (HR) chromosome images. High‐resolution images have clearer features and can better distinguish different chromosomes. Second, the size of the chromosome image is different originally, but the difference is even larger after processing. In order to meet the requirements of deep learning training, using simple image reconstruction inevitably affects the effect of super‐resolution (SR) network. Therefore, to ensure the effect of the super‐resolution network, we propose an image adaptive module that adapts the image to the same size as undistorted as possible. In addition, to solve the uneven distribution of all kinds of chromosomes, we propose the algorithm SMOTE [15] to generate a few classes that not only ensure the balance of the data but also solve the problem of slight overfitting due to random oversampling.

This paper analyses several chromosome classification methods and combines them with the SRAFBN to form a complete classification method called SRAS‐net. Experiments prove that our method is superior to existing advanced methods. Our main contributions can be summarised as follows:

1. In this paper, we propose an end‐to‐end chromosome classification method SRAS‐net based on deep learning. We use negative feedback network to generate high‐quality chromosome images before chromosome classification so that the images have more and clearer features. As far as we know, this is the first time that a super‐resolution network with a feedback mechanism is used for metaphase chromosome image classification.
2. When the image is processed for the super‐resolution network, its size is changed. Simply adapting to a uniform format significantly affects the effect of the super‐resolution network. Therefore, we propose an adaptive algorithm that resizes the images to the same size as undistorted as possible.
3. To solve the problem of inaccurate classification of sex chromosomes, we propose to use the SMOTE algorithm to generate a small number of sex chromosomes so that the model can learn more features of sex chromosomes.
4. We evaluate our method on a public chromosome dataset. Compared to current methods, our method shows superior performance, which is better than existing state‐of‐the‐art methods.

The structure of this paper is as follows: Section 1 gives a brief introduction to the background of the method proposed in this paper. Section 2 discusses research in related fields. Section 3 describes the methods used in this paper in detail and presents their theory and implementation. The experiment in Section 4 proves the effectiveness of our proposed method. Section 5 discusses the obtained results, analyses the shortcomings, and points out the direction for improvement. Section 6 summarises and draws conclusions.

## 2 RELATED WORK

Chromosome karyotype analysis is very important for prenatal diagnosis and genetic disease detection and has become a research focus in recent years. The purpose of karyotype analysis [16] is to determine whether chromosomes are abnormal or not. Chromosomal abnormalities are divided into two types: structural abnormalities and number abnormalities. Regardless of which type of abnormality it is, chromosomes must first be classified. The conventional method of chromosome classification is performed manually, which requires excellent technical quality but still cannot avoid the error caused by human subjective judgement. This method is time consuming and inefficient. With the development of computer technology, the automatic classification of chromosomes comes into being.

Ali et al. [17] proposed a method to use Support Vector Machines (SVM) to extract feature vectors corresponding to the features of each chromosome and then used these feature vectors to train an SVM classifier to classify chromosomes. This method is mainly based on the shallow features of the images and does not consider the deep features of the chromosome graphs, so the effect is not ideal.

Jindal et al. [18] proposed a chromosome classification method based on a Siamese network. In this method, SMAC and SPV were first used to straighten chromosomes, and then the Base‐CNN was used to pre‐train the Siamese network on paired chromosomes. Finally, MLP was integrated into the method and the final classifier was developed using BASE‐CNN and the pre‐trained Siamese network. This method first touches the deep features of the image, but due to the imperfect function of the classifier, the recognition accuracy still needs to be improved.

Qin et al. [19] proposed a new method called Varifocal‐Net to simultaneously classify the type and polarity of chromosomes. This method uses a G‐Net to extract global features of an image and an L‐Net to extract local features, and combines them with residual learning and multi‐task learning strategies to promote high‐level feature extraction. The results of evaluating 1909 karyotype analysis cases (87,814 chromosome images) showed that the method achieved 99.2% accuracy. This method introduces ensemble learning into chromosome classification covertly. Although the effect is very good, the dataset used has a great advantage in terms of quantity and resolution. When it is replaced by low‐resolution chromosomes, the effect is not ideal.

Monika et al. [20] proposed a chromosome classification method using a deep‐attention mechanism to learn chromosome banding features. They first proposed a residual convolutional recursive attention neural network that extracted chromosome band sequence features and then imported these sequences into a recurrent neural network (RNN) with the attention mechanism module applied to the upper and lower parts of the RNN. Due to the low quality of the data, the attention mechanism does not pay attention to the important features of the image, so this method only achieved a performance of 91.94% on the public BioImLab Q‐band chromosome dataset [21].

Lin et al. [22] proposed a chromosome classification method called MixNet. First, they developed a deep learning‐based chromosome classification system consisting of a chromosome encoding backbone and an adaptive header. Second, they developed the chromosome backbone using the aggregated residual architecture and proposed the adaptive header by aggregating pooling layers to classify latent chromosome features. This method achieved a classification efficiency of 96.50% on the public dataset.

Swati et al. [23] first proposed the use of a super‐resolution network for chromosome classification to improve the poor performance of low‐resolution chromosome classification. They used only a super‐resolution network consisting of three convolutional layers, and the experiment showed that the accuracy of this method increased from 91.80% to 92.36%.

Lin et al. [24] proposed a chromosome classification method called CIR‐Net, which proposed a simple and effective extension algorithm CDA based on Inception‐Resnet_V2 and could effectively solve the problem of automatically classifying clinical chromosome karyotypes with insufficient training data. This method achieved 95.98% performance on the clinical dataset (a total of 2990 chromosome images). However, the classification performance of sex chromosomes is significantly lower than that of autosomes.

Muna AI‐Kharraz et al. [25] proposed the ensemble learning method to average the results of three different classification models and obtained a classification performance of 97.01% on the public test set. Although this method achieved good results, ensemble learning is very time and resource consuming.

Zhang et al. [26] proposed a chromosome classification and straightening method based on an interleaved and multi‐task network. First, the method learnt multi‐scale features via an interleaved network. Second, high‐resolution features from the first stage were input into a convolutional neural network for chromosome linkage detection, and other features were fused and fed into two multilayer perceptron subnetworks for chromosome type and polarity classification. This method achieved a classification performance of 98.1% in their personal dataset. The method is also not ideal for sex chromosome classification.

Although the above method solves the chromosome problem to some extent, there are still some limitations [27, 28, 29, 30]. First, the image quality of the private dataset used in their method is very high. Considering limited hardware, not all hospital equipment can acquire high‐quality images. When applying their methods to low‐quality images, the performance is not very good [31, 32, 33, 34]. On the other hand, most of these methods have poor classification effects on sex chromosomes. For example, the method in Ref. [23] has an average accuracy of 95.89% on the public BioImLab dataset, but the classification accuracy for X chromosomes is only 86.84%.

## 3 PROPOSED METHOD

### 3.1 Architecture of the SRAS‐net

**FIGURE 2:**
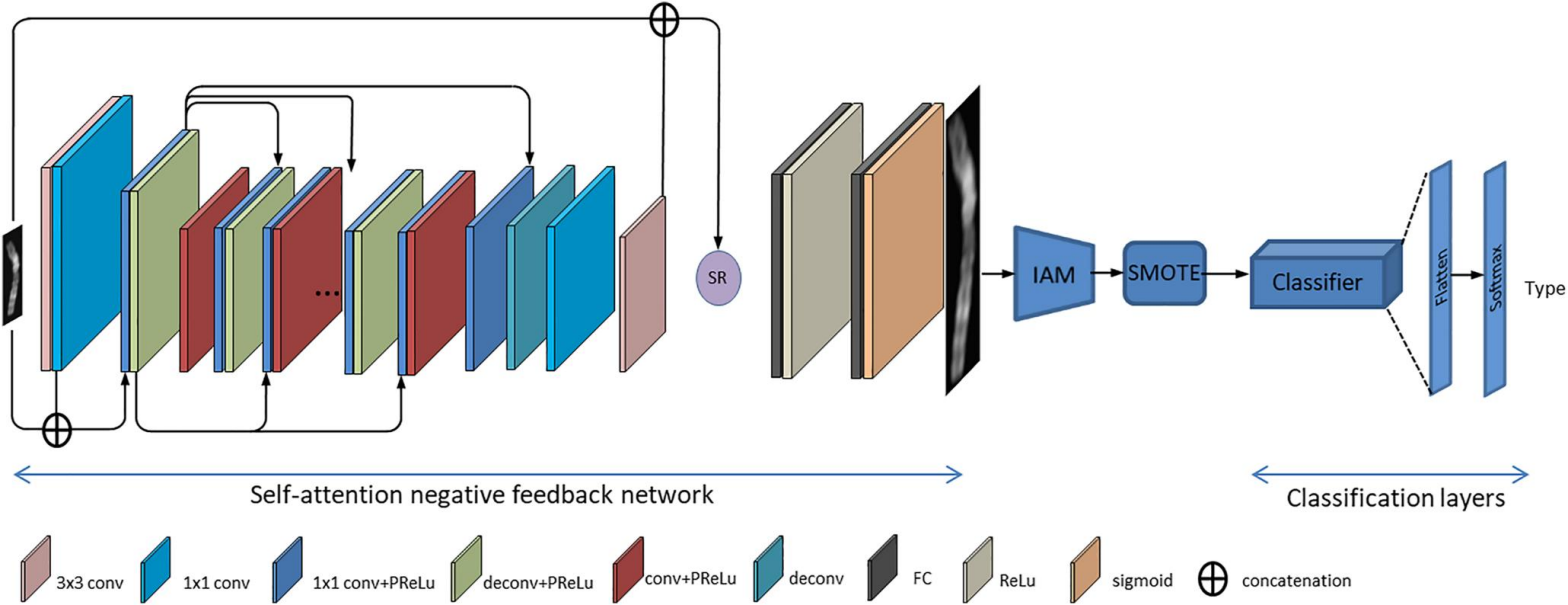
SRAS‐net network structure

As shown in Figure 2, the SRAS‐net network proposed in this paper mainly consists of three parts in addition to the basic classifier: (a) a negative feedback network was used to convert low‐resolution chromosomes into high‐resolution chromosomes; (b) an image adaptive module (short for IAM) was developed to fit images of different sizes to a uniform size without changing the information as much as possible to ensure the effect of SRAFBN and facilitate the training of the model; (c) the SMOTE algorithm is used to generate a small number of sex chromosome samples to ensure the balance of the number of samples while allowing the model to learn more sex chromosome features. The details of these three parts are described below:

### 3.2 Self‐attention negative feedback network

We know that the more details the image contain, the easier the classification and the better the effect of the classifier. However, when taking chromosome images, it is often difficult to obtain high‐resolution and detailed images. To solve this problem, we propose the use of the SRAFBN. This network model constrains the image mapping space and selects the key information of the image through the negative feedback mechanism to reconstruct the image, which is more real and clear and can better satisfy the human visual perception. This network model was proposed by us in our previous work, and the specific work and experimental details of the network can be found in the literature [14].

**FIGURE 3:**
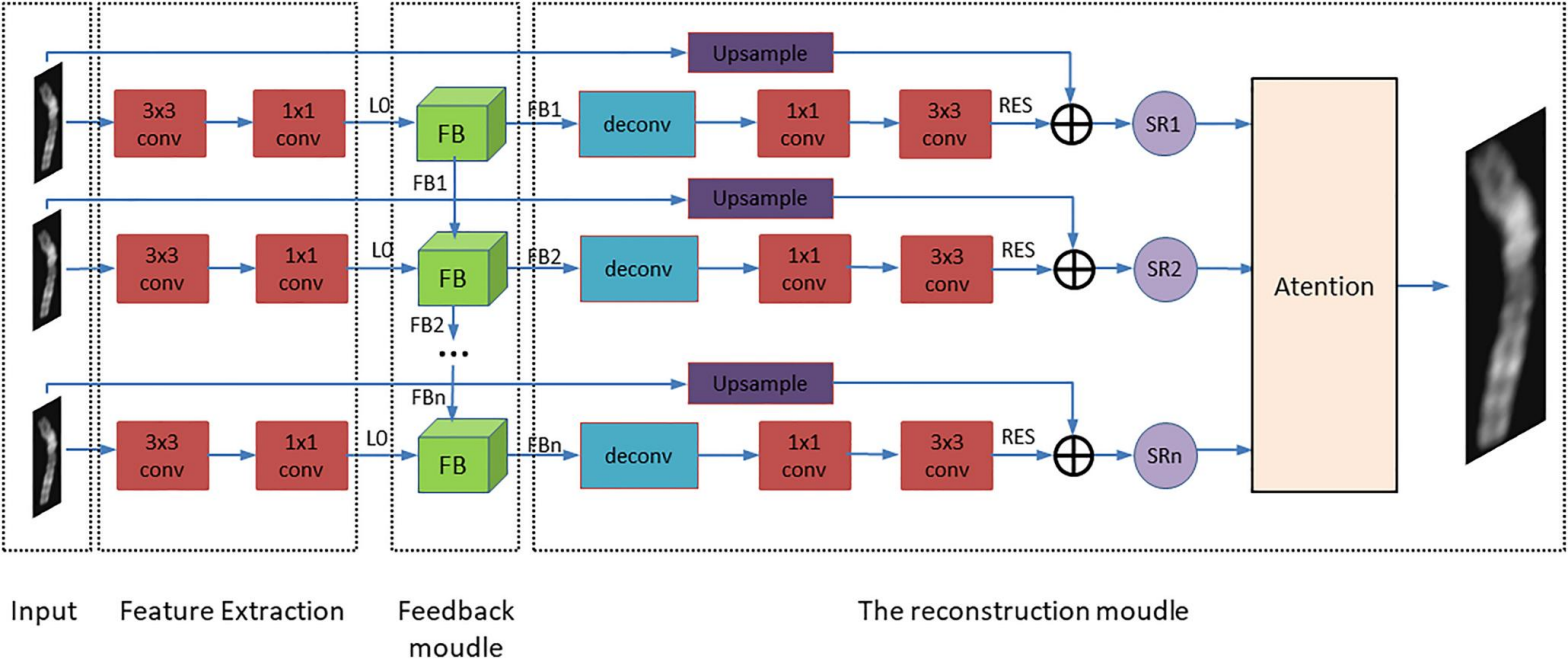
Self‐Attention Negative Feedback Network (SRAFBN)

Figure 3 shows the structure of the self‐attention negative feedback model. The entire network uses residual learning to solve the mapping relationship between the HR image and the LR image. It mainly consists of three modules: The first is a feature extraction module consisting of 3 × 3 conv and 1 × 1conv; the second is a negative feedback module (FB) based on a RNN; the third is a reconstruction module based on the mechanism of self‐attention.

In the feature extraction module, two convolutional layers are used to extract the shallow feature L_0 of the low‐resolution image LR, which serves as input to the negative FB:

(1) $$L_{0}=C\left(C\left(LR,K\left(3,3\right)\right),K\left(1,1\right)\right),$$

where *C* (∙) represents the convolution operation, and *K* (∙) represents the convolution kernel.

**FIGURE 4:**
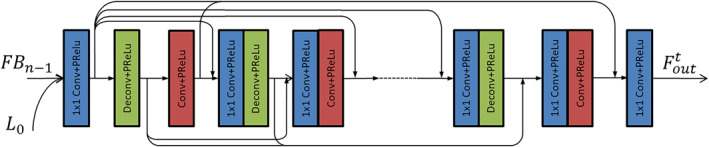
Negative feedback module (FB) structure

Figure 4 shows the specific operation of the negative FB. The network consists of several 1 × 1 convolutional layers for dimensionality reduction, coding convolutional layers, and corresponding decoding convolutional layers. The 1 × 1 convolutional dimensionality reduction layer is used to fuse feature images and perform dimensionality reduction operations; the encoding convolutional layer is mainly used to extract the abstract features of the image, and the decoding convolutional layer is used to recover the image details and generate the corresponding HR image. The encoding stack layer and the corresponding decoding stack layer are used to form a mapping group, and the output of the encoding build layer in each mapping group jumps to the input of the decoding build layer in all mapping groups by using the jump link line. Then the output of each mapping group decoding convolutional layer jumps to the input of all mapping group coding convolutional layers, and the dimensionality reduction of the channel number is performed by the 1 × 1 dimensionality reduction convolutional layer. By using the way of jumping connection, the input of the mapping group can be combined with the output information of all the previous mapping groups at the same time so that the problem of gradient disappearance caused by the increase of network layers is better solved and the optimisation of the network is promoted to a certain extent.

The entire module takes as input the shallow feature $L_{0}$ of the low‐resolution image and the image feature output by the previous module. The process can be formulated as follows:

(2) $$FB_{n}=\text{FBBD}\left(FB_{n-1},L_{0}\right),$$

where $FB_{n}$ stands for the image feature output by the negative FB after the nth iteration, and FBBD (∙) denotes the operation of the negative FB.

In the reconstruction module, in order to select the key information of the image, we introduce the self‐attention mechanism to assign different weights to the HR image output by each negative FB, so as to obtain higher quality images.

In the module, first, $FB_{n}$ passes through a deconvolution Deconv (∙), and then passes through two convolution *C* (∙), and finally generates a residual feature map consistent with the number of output image channels. The formula is as follows:

(3) $${\text{Res}}_{n}=C\left(C\left(\text{Deconv}\left(FB_{n}\right),K\left(1,1\right)\right)K\left(3,3\right)\right),$$

where ${\text{Res}}_{n}$ stands for the residual feature map generated after the nth negative feedback iteration of the low‐resolution image.

Next, add the up‐sampled image to it to obtain the iterative HR image. The formula is as follows:

(4) $$SR_{n}={\text{Res}}_{n}+\text{Upsample,}$$

where $SR_{n}$ is the HR image generated by the *n*th iteration of the negative FB, and Upsample is the HR image obtained by the low‐resolution image LR after double triple interpolation.

**FIGURE 5:**
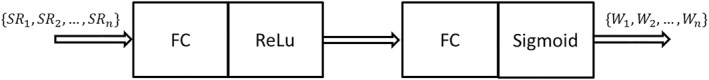
Self‐attention mechanism structure

After several RNN iterations, the self‐attention mechanism is used to give weight to the HR image SR generated in each iteration. Figure 5 shows the structure of the self‐attention mechanism, which takes SR generated in each iteration as input and obtains the weight of the SR image after training. The loss function (Loss) after each training is as follows:

(5) $$\text{Loss}=\left[{\text{loss}}_{1},{\text{loss}}_{2}\text{…},{\text{loss}}_{n}\right]\cdot W^{T},$$

(6) $${\text{loss}}_{n}=SR_{n}-LR,$$

(7) $$W=\left[W_{1},W_{2},\text{…},W_{n}\right],$$

where ${\text{loss}}_{i}$ stands for the loss function of ith RNN iteration, and W stands for the weight of the SR image generated *n* times.

At the end of the network training, the training parameters with the least losses are stored as models. In the testing process, the model extracts the high‐quality image generated by the last iteration of the RNN cycle as the final output.

**FIGURE 6:**
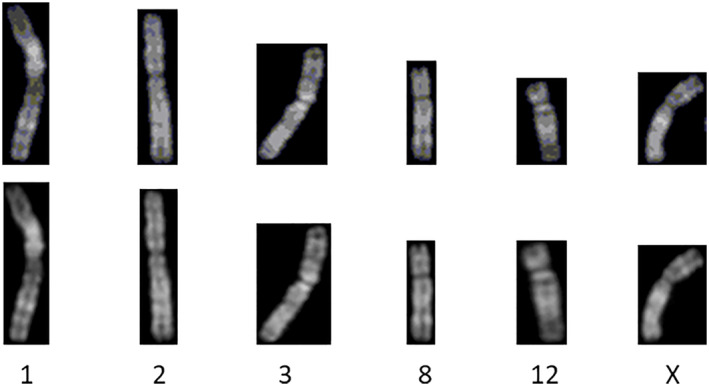
Comparison of the effect before and after the super‐resolution treatment

Figure 6 shows a before and after comparison of several chromosomes after SRAFBN processing. The number below the image represents the type of chromosome. From this, we can clearly see that the banding characteristics of all types of chromosomes have become clearer after SRAFBN processing, which is very helpful for solving the similarity problem between chromosomes.

### 3.3 Image adaptive module

We know that the sizes of the chromosome images segmented from the acquired cell images are different. After passing through the SRAFBN, the details increase and the size of the image becomes correspondingly larger. To adapt to the transfer learning operation in the following network, we need to resize the image to a uniform size. However, simply scaling the image will distort the image, change some important properties of the chromosome (such as the length, etc.), and significantly affect the effect of the SRAFBN. Therefore, we propose an algorithm that resizes each chromosome image to the desired size without affecting the chromosome image as much as possible and ensuring the integrity of the image information.

We use *X* = {$X_{i}$ |0≤*i*≤*N*} to represent the chromosome dataset processed by the SRAFBN, $X_{i}$ represents each chromatids, and *N* represents the number of chromosomes in the dataset. Then, the image adaptive module can be implemented as follows:

First, as shown in lines 6–21 in the following algorithm, we gradually approached the chromosome image to locate the upper, lower, left and right boundaries that contain all the information of the chromosomes:

(8) $$U=\min_{0\leq h\leq H}\left(X_{i}\cap E_{h}\neq 0\right),$$

(9) $$D=\max_{0\leq h\leq H}\left(X_{i}\cap E_{h}\neq 0\right),$$

(10) $$L=\min_{0\leq w\leq W}\left(X_{i}\cap E_{w}^{\prime }\neq 0\right),$$

(11) $$R=\max_{0\leq w\leq W}\left(X_{i}\cap E_{w}^{\prime }\neq 0\right),$$

where *U*, *D*, *L* and *R* stand for the upper, lower, left and right boundaries of the chromosome image $X_{i}$. $E_{h}$ stands for the sparse matrix in which hth row is all 1 and other rows are all 0. *H* stands for the height of $X_{i}$. *W* stands for the width of $X_{i}$.

As shown in line 22 in the following algorithm, after getting the boundaries, we crop the specific chromosome image:

(12) $$X_{i}^{*}=\text{Rect}\left(X_{i},\left(U,D,L,R\right)\right),$$

where $X_{i}^{*}$ stands for the cropping result of the chromosome image $X_{i}$, and Rect() stands for the cropping operation.

As shown in lines 23–27 in the following algorithm, we need to further adjust the image to within the threshold of the target size:

(13) $$I\left(X_{i}^{*}\right)=\left\{\begin{array}{l} X_{i}^{*},\;\max\left(H^{*},W^{*}\right)\leq 224\\ \text{resize}\left(X_{i}^{*},224/\text{max}\left(H^{*},W^{*}\right)\right),\;\max\left(H^{*},W^{*}\right)>224 \end{array},\right.$$

where *I* ($X_{i}^{*}$) stands for the adjusted chromosomal image, $H^{*}$ and $W^{*}$ stands for the height and width of $X_{i}^{*}$, and resize() stands for the image reproduction in a certain proportion 224/max($H^{*}$, $\;W^{*}$). The process Algorithm 1 is described as follows:

Algorithm 1 **Image Adaptation**

1

**Input:**

        Chromosome Dataset *X*={$X_{i}|0\leq i\leq N$};

        $E_{h}$ represents the sparse matrix in which hth row is all 1 and other rows are all 0.

**Output:**

        Processed new chromosome Dataset $\;X^{*}$.

1. **Function** *IAM*(*X*);

2. **for** $X_{i}$ **in** X **do**

3.         Convert each chromosome $X_{i}$ into array;

4.         Get the length H and width W of $X_{i}$;

5.         Initialise the upper, lower, left, and right boundaries *U*, *D*, *L*, and *R* of $X_{i}$ to ‐1;

6.         **for** w **in** W **do**

7.               **if** $X_{i}\cap E_{w}\neq 0$ and U== ‐1 **then**

8.                       U←w;

9.         **end if**

10. **      if** U ≠‐1 and $X_{i}\cap E_{w}=0$ **then**

11.               D←w;

12.       **end if**

13. **end for**

14. **for** h **in** H **do**

15.         **if** $X_{i}\cap E_{h}^{\prime }\neq 0$ and L== ‐1 **then**

16.                 L←w;

17.         **end if**

18.       **if** L ≠‐1 and $X_{i}\cap E_{h}^{\prime }=0$ **then**

19.               R←w;

20.       **end if**

21. **end for**

22. Cut $X_{i}$ according to the boundary *U*, *D*, *L*, *R*;

23. β←max(D−U,R−L);

24. **if** β > 224 **then**

25.         Reconstruct $X_{i}$ in proportion to 224/β;

26. **end if**

27. $X_{i}^{*}\leftarrow X_{i}\cup O_{224x224}$;

28.**end for**

29.**return** $X^{*}$.

30.**end Function**

This method allows the image to be resized while preserving the real information of the image as much as possible, which guarantees the effect of SRAFBN.

### 3.4 SMOTE

We know that a normal human has 22 pairs of autosomes and one pair of sex chromosomes. In females, the sex chromosomes are XX and in males they are XY. Thus, when a dataset of whole cells is segmented, there is inevitably an imbalance in the data of the X chromosome and the Y chromosome compared to the other autosomes. Through experiments (as evidenced in Part 4), we have found that the characteristics of the Y chromosome are more distinct. Although there are problems with the imbalance of the data, the effects are small. However, the size of the X chromosome is similar to that of #6–12 of the autosomes. When details such as centromeres and bands are not obvious, the disadvantages of data imbalance become more apparent. Therefore, we propose to use the SMOTE algorithm to solve this problem.

The SMOTE algorithm differs from the general method of random oversampling. It randomly selects a point on the two lines between the minority sample and its nearest neighbour sample to synthesise the minority sample. The specific steps of the algorithm are as follows:

We use $S_{\text{min}}$ to represent the sample set of the minority class, and then calculate the Euclidean distance from each minority class sample $X_{i}$ to other minority class samples, and select *K* samples with the shortest distance from them that is to obtain the *K* nearest neighbours of the minority class sample $X_{i}$. It is denoted as $N_{k}\left(X_{i}\right)$:

(14) $$X_{ij}^{*}={\text{argmin}}_{X_{ij}^{*}\in \left(S_{min}-X_{i}-N_{k}\left(X_{i}\right))\right.}\left(\sqrt{{\left(X_{i}-X_{ij}^{*}\right)}^{2}}\right),$$

(15) $$N_{k}\left(X_{i}\right)=N_{k}\left(X_{i}\right)\cup \left\{X_{ij}^{*}\right\},$$

where $N_{k}\left(X_{i}\right)$ is initially set as empty, and $X_{ij}^{*}$ represents the jth smallest sample of the minority sample $X_{i}$ in the set $S_{\text{min}}$, and the value range of *j* is 0 < *j* ≤ *K*. Equations (8) and (9) can be performed *K* times to obtain *K* nearest neighbours of the minority sample $X_{i}$. Then, set the sampling magnification *η* according to the unbalanced ratio of the sample:

(16) $$\eta =\frac{\left(\left|S_{\text{max}}\right|-\left|S_{\text{min}}\right|\right)}{\left|S_{\text{min}}\right|},$$

Then, for each minority sample $X_{i}$, randomly select *n* samples from its *K* nearest neighbours. For each selected ${X_{ik}}^{\prime }$, a new minority sample can be constructed:

(17) $$\;X_{\text{new},ik}=X_{i}+rand\left(0,1\right)\times \left({X_{ik}}^{\prime }-X_{i}\right),$$

where $\;X_{\text{new},ik}$ stands for a new sample constructed by each group of minority samples ($X_{i}$, $\;{X_{ik}}^{\prime }$ *^'*), and the value range of *k* is: 0 < *k* ≤ *η*.

After processing by the above algorithm, we can not only make the number of sex chromosomes the same as the number of other autosomes but also avoid the problem of easy over‐fitting caused by the random oversampling algorithm. This plays an important role when the subsequent classification model learns the characteristics of the sex chromosomes.

## 4 EXPERIMENTAL RESULTS

### 4.1 Dataset

To verify the effectiveness of the method proposed in this paper, we use the Bioimage Chromosome Classification dataset that has been published online to evaluate the performance of our proposed model. As shown in Table 1, the dataset was manually segmented, classified and labelled by cytologists from 119 normal human cells. The labelling of chromosomes 1–22 is 1–22, and the labelling of X chromosome is 23, and the labelling of Y chromosome is 0. The same optical microscope was used for the images, and all chromatids were polarised, that is, the short arm was at the top and the long arm was at the bottom. There were 46 chromosomes in each cell and a total of 5474 chromatids, including 193 X chromosomes, 45 Y chromosomes, and 238 × 22 autosomes from No.1 to No.22. We divided the images into a training set (4379) and a testing set (1095) in a ratio of 4:1 categories. Because the dataset is small, we treat one‐fifth of it as both the validation set and the testing set.

**TABLE 1:** Dataset description

No. of chromosomes	No. of cells (female/male)	Training datasets	Test datasets
5474	119 (74/45)	4379	1095

### 4.2 Evaluation metrics

In analysing of the experimental results, we pay particular attention to the accuracy of the image recognition results. Therefore, when verifying the performance of the method in this paper, we mainly use accuracy (Acc.) for evaluation. In addition, there are some other evaluation metrics [35]: precision, recall, $F_{1}-\text{score}$, confusion matrix etc. To calculate accuracy, we first need to calculate the following four basic indicators:

- True Positive ($TP_{i}$): The number of images in class *i* chromosome that are actually predicted as class *i*.
- False Positive ($FP_{i}$): The number of images in class *i* chromosome that are actually predicted as non‐class *i*.
- True Negative ($TN_{i}$): The number of images in non‐class *i* chromosomes that are actually predicted as non‐class *i*.
- False Negative ($FN_{i}$): The number of images in non‐class *i* chromosomes that are actually predicted as class *i*.

Then, for class *i*, the evaluation metrics after using the model classification can be expressed as follows:

(18) $${\text{Acc}}_{i}=\frac{TP_{i}+TN_{i}}{TP_{i}+TN_{i}+FP_{i}+FN_{i}},$$

(19) $${\text{Precision}}_{i}=\frac{TP_{i}}{TP_{i}+FP_{i}},$$

(20) $${\text{Recall}}_{i}=\frac{TP_{i}}{TP_{i}+FN_{i}},$$

(21) $$F_{i}=\frac{2\cdot {\text{Precision}}_{i}\cdot {\text{Recall}}_{i}}{{\text{Precision}}_{i}+{\text{Recall}}_{i}},$$

After calculating the accuracy of each type, Acc. of the whole model can be calculated as follows:

(22) $$\text{Acc}=\frac{\sum_{i}\left(TP_{i}+TN_{i}\right)}{\sum_{i}\left(TP_{i}+TN_{i}+FP_{i}+FN_{i}\right)},$$

Among them, the value of *i* ranges from 0 to 23.

### 4.3 Implementation details

The entire network was built based on the Tensorflow architecture. The image sizes are different. To meet the training requirements of the model, the image size is uniformly set to 224 × 224. When setting the hyperparameters, we set *batch_size* to 40 to balance memory efficiency and memory capacity. After many experiments, we find that the model essentially converges when the epochs reach 60–70, so we set the epochs to 100. To prevent the model from over‐fitting, we terminated the training early using the early stop method. The experimental data showed that the distance between the two extremes was not more than 21 epochs, so we set the patience value for early stop to 21. Also, we set the initial learning rate *lr* to 0.0005.

During training, we use the Adam optimiser to make the model better approximate the optimal value. In order for the dataset to meet the requirements of model training and to fully learn the properties of the chromosomes, we use an affine transformation to improve the dataset. Each chromosome image is rotated 24 times; each rotation is 15°. In addition, migration learning is introduced to use the already trained model to make the model converge faster. The indicator of early stopping is the accuracy of the verification set. The loss function used is the cross‐entropy loss function, which has always shown good performance. Also, before training the model, we randomly shuffled the training set and then input it to the network to reduce the random error.

### 4.4 Evaluation results

This section will evaluate the experimental results from both qualitative and quantitative aspects.

#### 4.4.1 Quantitative evaluation results

This section is divided into two parts. First, we compare our method with existing methods to verify the superiority of our method. Then, we evaluate the impact of SRAS‐net to analyse the performance of each of our modules.

##### Comparison with existing technology

We combine our method with several recent mainstream classifiers to form an end‐to‐end network structure and compare their performance with existing technologies on the same dataset by running the training process under the same benchmark. The results can be seen in Table 2. The first four methods have been proposed for chromosome classification in recent years and we used them to compare the performance of our method. Next is the performance of several popular classifiers on public datasets in recent years. Then, we investigate the effect of using these classifiers as a link to our method to form a new network structure. After comparison, it is found that the classification effect of the traditional classification model is generally low for low‐resolution chromosomes, which further encourages us to use the super‐resolution network to process chromosomes before classification. From the table, it can be observed that the network performs best when the classifier uses Inception_Resnet_V2, which is 97.55%. Therefore, we set the classifier to Inception_Resnet_V2 in the following analysis.

**TABLE 2:** Comparison of our method and existing methods

Method	Acc. (%)
Res‐CRANN [20]	90.42
Super‐Xception [23]	92.36
CIR‐Net [24]	95.89
MixNet [22]	96.50
Ensemble (VGG19, Resnet50, MobilenetV2) [25]	97.01
Resnet50 [36]	87.64
Xception [37]	91.80
VGG19 [38]	91.67
Inception_Resnet_V2 [39]	91.69
SRAS‐net (Resnet50; our proposed)	95.92
SRAS‐net (Xception; our proposed)	96.01
SRAS‐net (VGG19; our proposed)	96.56
SRAS‐net (Inception_Resnet_V2; our proposed)	97.55

##### Results evaluation

Table 3 is a comparison of the impacts before and after we added each functional module to the network in turn. At the beginning, we only used the backbone network Inception_Resnet_V2 to classify the dataset, and the classification accuracy reached 91.69%. Then, we use the SMOTE algorithm to generate minority classes, which improves the performance by 1.61%. It is worth mentioning that the X chromosome classification accuracy increased from 81.57% to 87.23%, which proves that the model using this method learns the X chromosome features better and effectively solves the problem of serious inaccuracy of X chromosome classification. If we do not process the image with IAM, the classification accuracy improves to only 96.83% compared to before. Finally, when we test our dataset with the complete SRAS‐net, the network achieves an average classification efficiency of 97.55%, confirming the effectiveness of super‐resolution processing for image classification.

**TABLE 3:** The model performance of adding each module in turn

Method	Acc. (%)
Inception_Resnet_V2	91.69
SMOTE + Inception_Resnet_V2	93.30
SRAFBN + SMOTE + Inception_Resnet_V2	96.83
SRAFBN + SMOTE + IAM + Inception_Resnet_V2 (SRAS‐net)	97.55

To observe the performance of SRAS‐net for each chromosome type, Table 4 shows the accuracy, precision, recall and F_1‐score for each chromosome type. As for accuracy, the classification performance of the proposed method is poor in No.4, No.8 and No.22, and the worst No.4 is 93.62%. However, it is worth noting that our method achieves 100% excellent results on No.1, No.2, No.6, No.15, No.16, No.21 and Y chromosomes. In addition, the classification effect of X chromosome also reached 97.87%, higher than the average accuracy, which further proves that our method can well solve the problem of serious inaccuracy of sex chromosome classification. In terms of precision, the proposed method performs poorly on chromosomes No.5, No.9, No.10, No.15, No.17 and No.19. The worst is that No.19 is only 92.16%, while the classification performance of the other categories is relatively good.

**TABLE 4:** Classification performance

Class (No.)	Acc. (%)	Precision (%)	Recall (%)	$F_{1}-score$ (%)
1	100.0	100.0	100.0	100.0
2	100.0	97.96	100.0	98.97
3	95.83	100.0	97.92	98.95
4	93.62	97.78	93.62	95.65
5	95.83	95.83	95.83	95.83
6	100.0	100.0	100.0	100.0
7	97.92	100.0	100.0	100.0
8	93.75	100.0	95.83	97.87
9	97.87	93.75	95.74	94.74
10	95.83	95.83	95.83	95.83
11	97.92	97.92	97.92	97.92
12	95.74	100.0	97.87	98.92
13	97.92	100.0	97.92	98.95
14	95.74	100.0	89.36	94.38
15	100.0	94.12	100.0	96.97
16	100.0	97.96	100.0	98.97
17	97.87	95.83	97.87	96.84
18	97.92	100.0	97.92	98.95
19	97.87	92.16	100.0	95.92
20	97.92	97.92	97.92	97.92
21	100.0	100.0	97.87	98.92
22	93.75	97.83	93.75	95.74
X	97.87	97.87	95.74	96.79
Y	100.0	100.0	100.0	100.0

**FIGURE 7:**
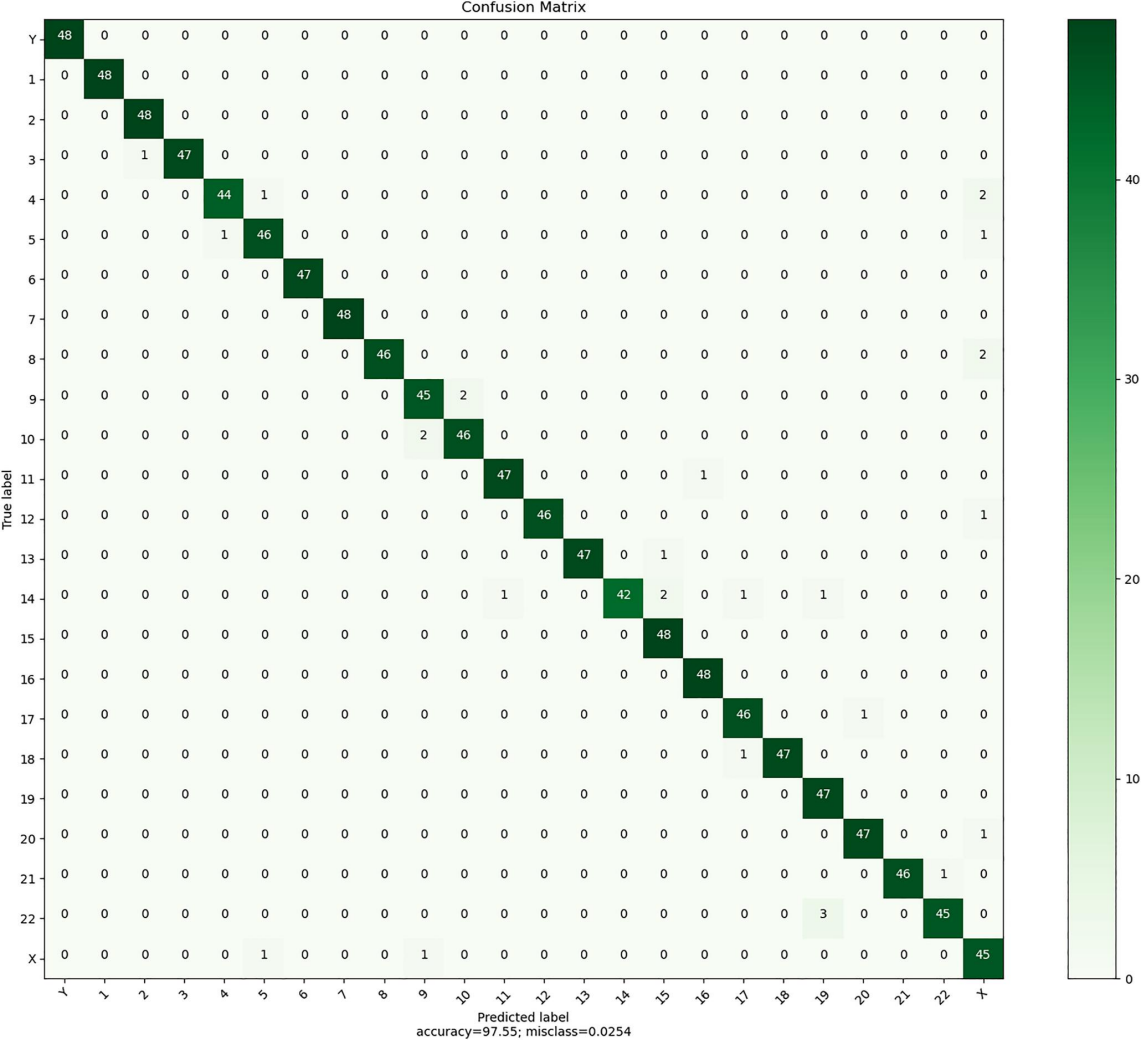
Confusion matrix

To further evaluate the performance of this method in chromosome classification, Figure 7 shows the confusion matrix. From the confusion matrix, it can be seen that most of the errors in chromosome classification are caused by the confusion of chromosomes of similar size. Overall, our method has a good performance in chromosome classification.

#### 4.4.2 Qualitative evaluation results

**FIGURE 8:**
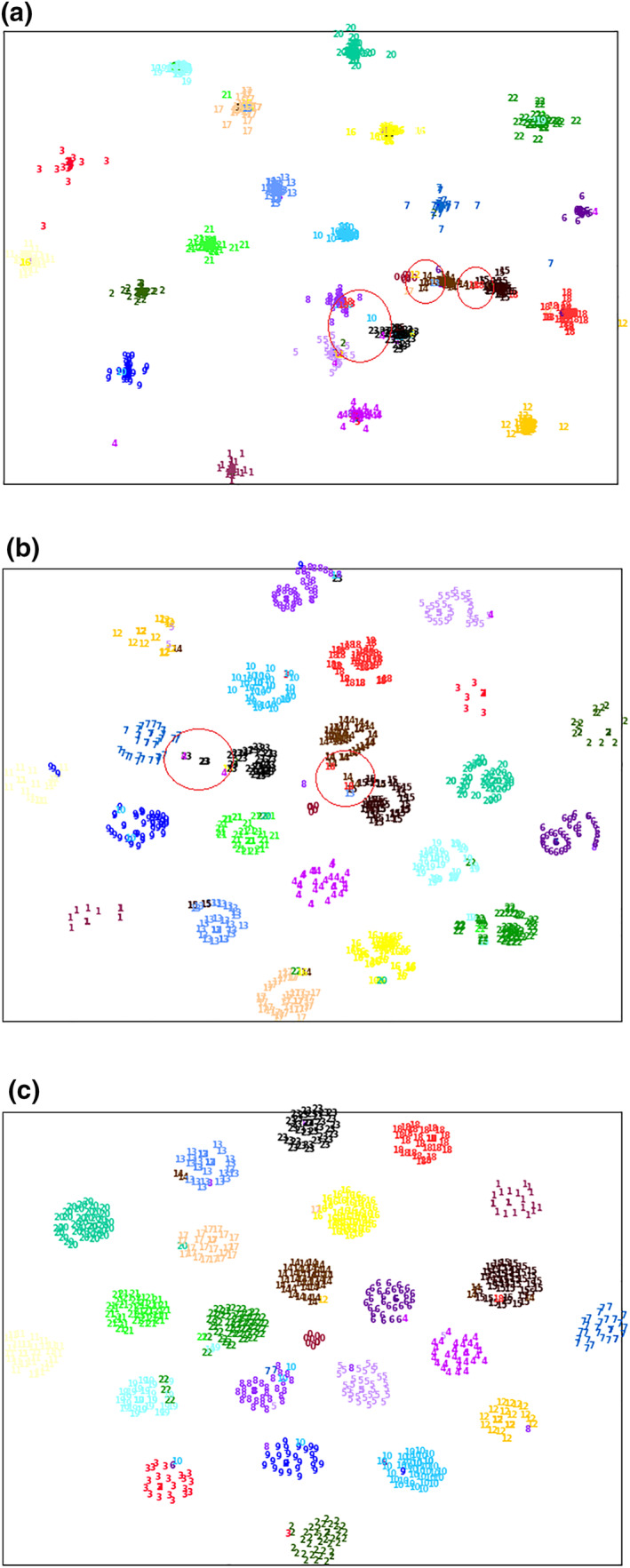
Use the T‐SNE method to display the test set features extracted by the network. (a) Represents the visual features of the test set classified using Inception_Resnet_V2. (b) Represents the visual features of the test set classified using CIR‐Net. (c) Represents the visual features of the test set classified using our method

To intuitively evaluate the classification performance of the network, we extracted the chromosome features trained on the training set from the middle layer of the network and evaluated the classification effect of the network on the test. We apply the T‐SNE [40] method to the feature display of the test set, reducing it from high‐dimension to visualised two‐dimension. As shown in Figure 8, the test set is clustered by category. The numbers 1–22 represent 22 autosomes, the number 0 represents the Y chromosome, and the number 23 represents the X chromosome. The more obvious the separation between different clusters, the better classification effect the network has. As you can see from the figure, Inception_Resnet_V2 is able to classify chromosomes well, but the classification between Y chromosome, chromosome 14 and chromosome 15 and between X chromosome and other chromosomes is still insufficient. When CIR‐Net is used for classification, the above problems have been significantly improved, but the problems still exist. However, when using our method for classification, except for the inaccurate identification of a few individual chromosomes, different chromosomes can be well separated and accurately identified overall.

## 5 DISCUSSION

The above experimental verification shows that our method performs well in classifying low‐resolution chromosomes and improves the classification efficiency of sex chromosomes. The reason for this good performance is that we use the SRAFBN to convert the low‐quality chromosome image into a high‐quality chromosome image. We know that the higher the quality, the clearer the features of the image. In a classification task, it is easier for the classifier to recognise and the classification effect is better. To solve the problem of inaccurate identification of the sex chromosome, we use the SMOTE algorithm to generate a small number of sex chromosome samples so that the number of samples is balanced and the model can learn more sex chromosome features to achieve a better classification effect.

Although our method has achieved good results in low‐resolution chromosome classification, there are still problems that need to be investigated. The poor classification of chromosomes is mainly caused by the interference of chromosomes of the same group or adjacent groups of similar size. For example, chromosome 22 is a short acrocentric chromosome that belongs to group G and is severely affected by chromosome 21, which belongs to the same group and chromosome 19, which belongs to the adjacent group.

We also note that our method is generally superior to existing advanced methods but slightly inferior to them in classifying some individual classes. In recent years, both Varifocal‐net [19] and Muna AI kharraz et al [26] have used the ensemble learning method, which shows lower degradation in type classification, suggesting that ensemble learning may be a method to improve universality. Moreover, Zhang et al [25] used multimodality in their method to fully learn the multiscale features of chromosomes. Considering the features of chromosomes, we will use a two‐stage classification method in the future. First, we will classify them into seven categories according to the international classification standard, and then we will classify them into corresponding categories according to the dumb features of chromosomes.

## 6 CONCLUSION

Considering that high‐resolution chromosomes are not available, a classification method SRAS‐net suitable for low‐resolution chromosomes is proposed in this work. The method is divided into three stages: First, a SRAFBN is used to convert low‐resolution chromosomes to high‐resolution chromosomes. Then, the IAM is used to bring the chromosome image to the same size as much as possible without distortion, which further enhances the effect of the SRAFBN. We use the SMOTE algorithm to generate a small number of sex chromosome samples to ensure a balanced number of samples while allowing the model to learn more sex chromosome features to achieve better sex chromosome classification. Finally, we use Inception_Resnet_V2 to classify the chromosomes. The experimental results show that our method achieves an average classification effect of 97.55%, which is better than the existing advanced methods. Also, the problem of inaccurate classification of X chromosomes was well solved, which encourages us to first use the super‐resolution network in chromosome classification to process chromosome images and achieve better results.

## CONFLICT OF INTEREST

The authors declare that they have no commercial or associative conflict of interest in connection with the work submitted.

## Data Availability

The data that support the findings of this study are available from the corresponding author upon reasonable request.
